# Progress along developmental tracks for electronic health records implementation in the United States

**DOI:** 10.1186/1478-4505-7-3

**Published:** 2009-03-16

**Authors:** David W Hollar

**Affiliations:** 1 School of Medicine, The University of North Carolina, Chapel Hill, USA

## Abstract

The development and implementation of electronic health records (EHR) have occurred slowly in the United States. To date, these approaches have, for the most part, followed four developmental tracks: (a) Enhancement of immunization registries and linkage with other health records to produce Child Health Profiles (CHP), (b) Regional Health Information Organization (RHIO) demonstration projects to link together patient medical records, (c) Insurance company projects linked to ICD-9 codes and patient records for cost-benefit assessments, and (d) Consortia of EHR developers collaborating to model systems requirements and standards for data linkage. Until recently, these separate efforts have been conducted in the very silos that they had intended to eliminate, and there is still considerable debate concerning health professionals access to as well as commitment to using EHR if these systems are provided. This paper will describe these four developmental tracks, patient rights and the legal environment for EHR, international comparisons, and future projections for EHR expansion across health networks in the United States.

## Background

Factors that have contributed to the United States' drive for electronic health records systems include a growing, more diverse American population, increasing socioeconomic and health disparities, a significant percentage of Americans who have no health insurance and the lack of socialized medicine or "universal" insurance coverage, a distributed medical system that emphasizes specialization over general practice in predominantly urban areas, poorer per capita health cost-benefit ratios, and a high incidence of medical errors [1-5]. With respect to the last point, The Institute of Medicine (IOM) estimated that between 44,000 and 98,000 Americans die each year due to preventable medical errors [2].

During July, 2003, a national consensus conference of over 100 leading American health professionals, coordinated by the U.S. Department of Health and Human Services, identified the core components, goals, and mechanisms for implementing a National Health Information Infrastructure (NHII; see List of Abbreviations) with a corresponding National Health Information Network (NHIN) [6]. The conference agreed that efforts at achieving comprehensive linked electronic health records (EHR) and the NHII/NHIN would require public-private partnerships and should exhibit: (a) data security, (b) common standards, (c) a non-proprietary nature, (d) national scaling, (e) incremental growth, (f) simplicity in structure, (g) low entry barriers, (h) support of distributed systems, (i) flexibility and responsiveness, and (j) use of standard Internet protocols [6]. This conference and a confluence of other efforts (described below) have served to drive a strong national movement towards widespread use of EHR. Official federal endorsement of these efforts came in 2004, when U.S. President George W. Bush appointed a national coordinator for Health Information Technology, Dr. David Brailer (seceded by 2009 interim coordinator Dr. Robert Kolodner), started the adoption of federal standards for national information technology infrastructure, and announced that the nation's medical records systems would become paperless within ten years [7].

While this ten-year goal is most likely unrealistic, these efforts promise to develop electronic health records for every American that would be transferable to any health services provider. Furthermore, with increasing healthcare disparities and the 2008–2009 global economic crisis, U.S. President Barack Obama signed into law the American Recovery and Reinvestment Plan of 2009 on February 17. Title IX (Labor, Health and Human Services, and Education) of this $787 billion (U.S.) plan includes $20 billion to implement electronic health records systems and to train healthcare workers to use these systems [8].

To date, the development of such records has proceeded along four primary tracks: (a) Development of immunization registries and linkage with other health records to produce electronic child health profiles (CHP), (b) Local and regional hospital system demonstration projects to link together patient medical records, (c) Insurance company projects linked to ICD-9 codes and patient records for cost-benefit assessments, and (d) Consortium groups sharing experiences and modeling of systems requirements and standards for data linkage. This paper will describe these four developmental tracks, the legal environment/implications of their implementation, and future projections for their expansion across health networks in the United States.

### Child Health Profiles (CHP)

Substantial progress has been made in the linkage of children's electronic health records, especially given the long-term medical and public health focus on improved children's health during the 20^th ^century. Between 1992–2004, the Robert Wood Johnson (RWJ) Foundation funded the Public Health Informatics Institute (PHII; Decatur, Georgia) with over $30 million (U.S.) to improve electronic systems for storing and transmitting immunization records between public health departments and health care providers at state and national levels. This funding continued with efforts to link these immunization records to other health registries, thereby creating an electronic Child Health Profile (CHP) [9]. Projects were further supported by simultaneous funding of state public health departments by the U.S. Health Resources Services Administration (HRSA, a division of the U.S. Department of Health and Human Services) [9,10].

#### CHP Need

A specific focus of the CHP projects involved children with special health care needs (CSHCN), operationally defined by the HRSA Maternal and Child Health Bureau as being "those children who have or are at increased risk for a chronic physical, developmental, behavioral, or emotional condition and who also require health care-related services of a type and amount beyond that required by children generally" [[11-13,11], p. 138]. These children include children with genetic or metabolic conditions, birth defects, and other disabilities.

CSHCN represent approximately 12.8% of American children, and roughly 20% of American households with children include a CSHCN [12,13]. Furthermore, 9.73% of CSHCN experienced delayed or forgone care, with such lapses in healthcare being significantly associated with race (i.e., highest for Hispanics), age (i.e., being an adolescent CSHCN), region (i.e., living in the American South or West), having severe functional limitations, being at or near the federal poverty level, and having no medical insurance [13]. Additionally, the two most frequently cited reasons for delayed care were financial problems (including transportation issues) and provider non-accessibility, both of which were significant across the same pattern of associations for general delayed/foregone care [13]. Financial and transportation issues are also of particular note since they are listed barriers to be addressed in the federal *Healthy People 2010 *[14] objectives for improved American healthcare.

The value of data linkage for NBS into a CHP for CSHCN that can be followed up by health services providers is forcefully argued by Hinman et al. [9], who estimated the annual number of missed cases of classical phenylketonuria (n = 10) and congenital hypothyroidism (n = 52), out of 4,058,814 U.S. births [15,16]. They based these estimates upon the number of children screened for these conditions, numbers of true and false positive cases, and number of cases that were lost to follow-up testing and potential treatment [9]. The lifelong medical, psychological, quality of life, educational, and financial impacts of each missed phenylketonuria case on the patient, their family, and society are substantial [17].

Furthermore, Desposito et al. [18] found that 31% of surveyed pediatricians reported receipt of NBS-positive results more than 10 days after testing was completed, and 28% of respondents fallaciously viewed no results as indicating a negative screen not requiring follow-up, thus potentially complicating the lack of urgent care that might be needed by some of these newborn infants.

Therefore, the combined PHII, RWJ, and HRSA effort to create electronic child health profiles directly responded to the American Academy of Pediatrics Newborn Screening (NBS) Task Force recommendations that child health delivery required an adequate systems infrastructure that links the NBS heel-stick program (for identifying genetic or metabolic conditions in newborns) with birth registration, immunization, newborn hearing screening, and the Women, Infants, and Children (WIC) support programs [10,19]. The electronic Child Health Profile would enable rapid identification of conditions in CSHCN that lead to swift follow-up services for confirmed conditions.

#### CHP Results

HRSA awarded seven states (i.e., Arizona, Colorado, Iowa, Massachusetts, Missouri, Rhode Island, and Wisconsin) with the first HRSA SPRANS State Development Grants for Newborn Screening Efforts and Infrastructure Development [10]. Fifteen more states were funded for such exploratory programs in 2000–2001, and of these 22 states, 16 states would receive further funding to actually implement Child Health Profiles [9,10]. Rhode Island, Missouri, Oregon, and Colorado were successful at early data linkage projects that created functioning CHPs. Other states, such as Tennessee with its TN-CHP program, utilized unique public health-university-advocacy group partnerships that specialized in data for children with genetic/metabolic conditions [20].

By 2001, the PHII and HRSA state immunization programs had yielded highly successful results, with many states showing dramatic improvements in the numbers of newborn infants receiving at least one immunization and the numbers of children receiving the recommended array of childhood preventative vaccinations [21,22]. Nationally, 89.4% of children aged 19–35 months had received polio vaccinations, 76.3% had received varicella vaccinations, and 73.7% had received the 4:3:1:3:3 series of DTP (Diphtheria, Tetanus, Pertussis), poliovirus, measles, Haemophilus influenzae b, and Hepatitis B immunizations [21].

Nevertheless, a focus on newborn screening (NBS) continued because of expanded technological capacities (i.e., the invention of tandem mass spectrometry, TMS) for evaluating dozens of genetic and metabolic conditions [23]. During early 2005, only seven states were screening every newborn child for more than seven genetic or metabolic conditions. However, by late 2006, 25 states were screening more than 20 conditions using TMS [24]. Traditional genetic and metabolic conditions tested since the 1970's had included phenylketonuria, galactosemia, congenital hypothyroidism, and hemoglobinopathies (e.g., Sickle Cell Anemia, Thallasemia). TMS enables blood serum measures of levels and ratios of the 20 biological amino acids and other biochemicals, thus expanding the measurement array to over 50 conditions. Unfortunately, the incidence of many of these conditions is unknown, and many conditions have no known treatment [24].

Therefore, the state CHP programs were highly successful at increasing childhood immunizations. Nevertheless, NBS and TMS were creating an exponential data explosion. State CHP projects were successful at linking together many children's health records, but they encountered logistical issues with the datasets. These problems included varying computer language formats for databases, inconsistent child and family identifiers for data linkage, and political (e.g., "data silo") resistance. Problems varied from state to state, but all states faced data linkage barriers.

#### Model CHP Programs

One of the most successful CHP programs has been Rhode Island, which currently has over 150,000 children in its Kidsnet database, with approximately 14,000 new births each year, nine different linked databases, and a majority of health care systems and private physicians utilizing the linked data [25]. Rhode Island has carefully implemented its program since 1997 and currently serves as the national model for an electronic Child Health Profile.

Including Rhode Island, 12 of the 22 HRSA/PHII-RWJ funded state projects were actively integrating child health records by 2004, six other states were still in the planning stages, and four states were no longer planning because of a lack of political and financial support from their state governments [26]. Thirteen states had linked NBS and dried blood spot data, nine states had these two databases linked with vital registration, five states had these three databases plus an immunization registry, and nine states had no immunization registry included at all [26]. The reporting state programs cited organizational constraints (13 states), a difficult external political environment (ten states), financial resources (11 states), data sharing agreements (nine states), and data duplication (six states) as major challenges to their projects [26].

Based upon Rhode Island's success, public health leaders there and PHII identified central features of successful electronic health records [27]. Nineteen core principles of EHR include: making information available to parents, families, providers, and programs; involving these stakeholders in the system design; maintaining security and confidentiality of individual patient data; ensuring timeliness of data availability using appropriate technologies; using computerized audit trails of who accesses data; making the system simple to use and adaptable to changing technology; making the system cost effective; and most importantly, preventing use of the data for punitive or discriminatory purposes. Patient/parental control of the data is a unique component of the Child Health Profile approach, although this latter goal may not be achievable in an NHII given the strong involvement of for-profit service providers and insurance companies, an issue that will be addressed below. PHII [27] also identified 22 core functions of EHR, many of these functions mirroring the 19 principles, but specifically advocating the establishment of a record for each newborn within 2 weeks but ideally within 24 hours of birth (a critical point for potentially life-threatening genetic or metabolic conditions such as classical galactosemia [28-30], establishing unique identifiers for each patient in the database; retrieving and processing immunization and hearing data within one month of service and NBS dried blood spot within 24–48 hours; allowing provider data entry at patient visit and tracking of individual case progress as well as immunization updates throughout the treatment process; and using national standards for electronic data exchange [27]. Furthermore, PHII operationally defined the purpose of the electronic Child Health Profile "to facilitate assessment and prompt provision of appropriate services to ensure an optimal healthy start for all children and improve the health of children" [[27], p. S54].

D'Alessandro and Dosa [31] reiterated the child and family orientation of the Child Health Profile that is the centerpiece of the Rhode Island Kidsnet and PHII programs. The current international healthcare movement from traditional medical to bio-psychosocial philosophical service delivery models is easily extended into the new information technology facet of healthcare delivery, where patient empowerment is a central theme [31].

#### Prospects for CHP

An electronic Child Health Profile can be used to improve developmental tracking and service provision to CSHCN and their families. It can serve as an integrating healthcare tool for improving individual access to *the medical home*. It can also allow primary providers to monitor patient improvements and treatments over time regardless of the location of service provision [32]. The integration of service delivery can strengthen patient and family access to medical homes, thereby improving patient safety and services received [33,34].

A 1998–2000 survey of families with CSHCN, conducted by Family Partners/Family Voices and Brandeis University, found that families were receptive to the idea of having their child's records in an electronic Child Health Profile, although there were concerns over data security, confidentiality of records, and authorized access to the records [35]. One parent said she was fortunate her daughter with a metabolic condition was born in a European country where the health laboratory operated continuously, unlike their U.S. home in a "predominantly rural state" [[35], p. S26]. While American NBS programs with Tandem Mass Spectrometry (TMS) are beginning to operate around the clock, states differ with respect to newborn screening tests offered, and no state has 100% systems integration for follow-up. Even so, Hinman et al. [9,27] argued for the rapid integration of systems to increase follow-up services. They also argued that the data quality does not have to be perfect, meaning that the occurrence of more false positives will not harm the process of providing improved healthcare [27]. Nevertheless, some experts have questioned the potential psychological harm done to families of false positive children in NBS [36,37]. Given the exponential increase in the amount of information being gleaned from TMS and newborn screening [37,38], some researchers have suggested that reporting of presumptive positives from TMS proceed more cautiously than at present [37].

Wild and Fehrenbach [39] and Wild et al. [40] highlighted best practices for the use of children's health information in two CHP development products designed to guide programs that are implementing linked electronic records: *Integration of newborn screening and genetic service systems with other maternal & child health systems: a tool for assessment and planning*, and the accompanying *Tool for Assessment and Planning*, both available from .

Besides providing rapid follow-up services to child well-being and establishing a medical home, the aggregate data obtained from the Child Health Profile or EHR include regional and national assessments of health care delivery to mothers and children [41-43], the importance of which includes addressing regional variations in the prevalence of conditions and diseases as well as addressing the problem of health disparities based upon race, socioeconomic differences, and urban/rural availability of service providers. The logistical and technical problems encountered by the various state projects described are reiterated in the more comprehensive regional demonstration projects described below. Additionally, service providers, particularly private physicians in rural areas, experience variations in the level of access to information technology, willingness to learn and invest in such technology, and availability of information technology experts to maintain the technology [44].

### Regional Hospital Information Organizations (RHIOs)

Two regional hospital information organization (RHIO) projects have emerged as leading models for multi-system data linkages across all aspects of healthcare and human lifespan: (a) the Regenstrief Institute (Indianapolis) Medical Records System, and (b) the Volunteer e-Health Initiative (Memphis).

#### Regenstrief RHIO

The Regenstrief Institute provides the national model program for comprehensive integration of hospital records [45], having started as a computerized patient management system for outpatient diabetes care in 1972. Over the ensuing decades, this integrated information system expanded to every aspect of patient care, and it now serves nine counties in the metropolitan Indianapolis region. The Regenstrief RHIO embraces five interconnected major hospital systems, all county and state public health departments, 3,000 specialists, 30 public school clinics, and provides services for 2.5 million clinic, 390,000 emergency department visits, and 165,000 inpatient visits each year [45]. This Indiana Health Information Exchange has developed data sharing agreements between all participating providers within the contexts of federal laws (i.e., HIPAA and FERPA, described below) to produce a system of "federated data vaults" that are housed at the Regenstrief Institute [45,46]. Each service provider loads its own data vault using secure, encrypted electronic transmissions using the HL7 clinical messaging standard (Health Level Seven, Inc., Ann Arbor, Michigan). Most importantly, each service provider has administrative authority over its own data vault.

Besides HL7, other data standards being used in the Regenstrief system include LOINC codes for laboratory results, CPT-4 codes for clinical procedure names, ICD-9 diagnoses, National Drug Codes and RxNorm codes for medications [45,46]. These standards are also endorsed by the United States Government's electronic health information standards. At the Regenstrief Institute, data from the many collaborating service providers are transmitted to respective data vaults, and the data are matching and linked using deterministic and probabilistic fuzzy matching algorithms (e.g., name, social security number, birth date, and gender) to produce a centralized global patient registry linking data from the respective vaults [45-47].

#### Volunteer e-Health RHIO

The Volunteer e-Health Initiative [[47]; ] is modeled after the Regenstrief Institute project. Volunteer e-Health capitalizes on the national EHR efforts, particularly NHII/NHIN, while specifically addressing a health-related fiscal crisis at the state level. The state of Tennessee provides a combined federal Medicaid and state-funded health insurance program called TennCare to citizens who cannot afford private health insurance. Medicaid was created by the United States Congress in 1965 under Title 19 of the Social Security Act, and it provides joint funding for state-controlled health insurance programs serving the poor, older adults, and persons with disabilities. TennCare, Tennessee's version of Medicaid, was providing health insurance to about 25% of the state's 5.8 million residents by 2004. Unfortunately, the program was straining the state's budget, primarily due to its extensive array of coverage and lack of caps on pharmaceutical benefits [47].

As recently as 2004, Tennessee ranked poorly among the fifty U.S. states on several indicators of child and adolescent health, including low birthweight babies (45^th^), infant mortality rate (44^th^), and percent of children without a parent who had full-time employment (43^rd^) [48]. Volunteer e-Health selected a three-county area (urban Shelby county, rural Fayette and Tipton counties) comprising the greater city of Memphis metropolitan area, with 18% of all TennCare beneficiaries living in Shelby county alone (out of 95 counties and five metropolitan areas in Tennessee). The project, directed by the Tennessee state Department of Finance and subcontracted to Vanderbilt University's Center for Better Health, received approximately $10 million matched between the state and the U.S. Agency for Healthcare Research Quality (AHRQ, a division of the U.S. Department of Health and Human Services). The five-year project mission seeks to use the Vanderbilt University Medical Center Star-Chart medical records system as a Regenstrief-style data vault system for matching, linking, and de-duplicating health records systems of approximately one million Tennesseans seen at 12 major hospitals and clinics in the Memphis greater metropolitan area, including institutions such as Baptist Memorial Health Care, Lebonheur Children's Medical Center, Methodist Healthcare, St. Jude Children's Research Hospital, and the University of Tennessee Medical Group [47].

The Volunteer e-Health Initiative utilizes data sharing agreements between the partnering medical facilities, control over each data vault maintained by the host medical facility, and the use of Consolidated Health Informatics [47,49,50] adopted standards (e.g., Health Level 7 messaging standards; NCPDP SCRIPT for retail pharmacy transactions; LOINC for laboratory tests, orders, results; SNOMED CT for diagnoses, etc.). The project utilizes a data matching, linking, and de-duplication approach similar to that of Regenstrief and other EHR projects. Furthermore, it is expanding its collaboration activities to assist medical center projects in other southern states (e.g., Florida).

Project director Dr. Mark Frisse [51] emphasized the prevailing need to incorporate business models [52] into EHR in order to address the American healthcare crisis and specifically to reduce the inefficiencies prevalent in managed care, a common theme in EHR efforts. In championing Volunteer e-Health, Tennessee Governor Phil Bredesen has extensive healthcare management experience and has emerged as a national leader in the health information technology (HIT) movement [53]. Nevertheless, the TennCare crisis is further clouded by the removal of over 100,000 Tennesseans from coverage, capping of pharmaceutical benefits, and institution of an alternative state-covered, low-cost insurance plan that addresses preventative health care and short hospital visits, but not catastrophic care [54]. Whereas the Volunteer e-Health Initiative is not connected with the controversy surrounding the TennCare cuts and the new alternative insurance program for the poor, the two entities represent different, complementary aspects of a comprehensive strategy to provide health care to as many people as possible while addressing escalating healthcare costs at the state level. Furthermore, it further highlights the increasing number of Americans (currently estimated at 47 million) who cannot afford health insurance [55,56] and the close alignment of private health insurance companies with the electronic health records movement. It should also be noted that, in Tennessee, under his executive order 35 (June 1, 2006), Governor Bredesen appointed an e-Health Advisory Council to oversee these efforts, with the 18-member council consists of six business leaders, five health care administrators, three insurance company representatives, one academic, and one hospital chief medical officer [57].

While the Regenstrief and Volunteer e-Health RHIOs are following a common model, many hospital systems across the United States have developed their own systemic electronic health records systems that comprehensively link information for all patients who visit their service locations. For example, UNC Health Care links patient data for all of its component hospitals, affiliate hospitals, clinics, nursing homes, and the North Carolina Department of Public Health . The Robert Wood Johnson Foundation [58] reported that only 5.4% of medical centers had a fully functional EHR, 15.1% had a basic EHR, and 79.5% had no EHR. A critically important EHR task that the Volunteer e-Health RHIO addresses is inter-system communication, albeit via the imposition of an external system on collaborating health systems.

### Insurance Company Involvement in EHR

While Tennessee is but one of many similar cases across the United States dealing with the difficult complexities of EHR and the overall context of escalating healthcare costs in the absence of socialized medicine, it illustrates minimally peripheral involvement of private insurance companies in these electronic health records efforts. Nevertheless, health insurance companies have an intensive interest in EHR, for obvious cost effectiveness reasons and the avoidance of "unneeded" medical procedures, however defined. Insurance companies are involved in EHR development activities at a level comparable to the children's and hospital system models described above. Still, their activities are poorly documented, so their success in addressing infrastructure problems such as data matching, unique identifiers, and de-duplication of records are rarely presented, and the public health research literature has little documentation on these activities to compare progress with federal and state-funded programs, which obviously for the latter must be presented in order to be answerable to citizens, in contrast to private business, including insurance companies.

Whereas state and demonstration projects funded by federal and state sources have received much of the spotlight for EHR, private insurance companies have been extensively involved with EHR activities, both independently and in collaboration with the national/state projects. Insurers have a vested interest in EHR given that they are intricately involved in healthcare provisions, and they have a responsibility to their patient clients, their shareholders, and their own fiscal survival to provide optimum healthcare at affordable prices. The business models for healthcare, championed by Frisse and Governor Bredesen in Tennessee among many other leaders, recognize a need to strike a balance between optimum and cost-effective health care [51-53]. Whereas insurers may be viewed negatively by consumers given deductible expenses, ever increasing insurance premiums for self-employed individuals and small businesses versus large employers and state worker subsidized health plans for large-scale working groups, and even dramatic state-to-state level variations in health insurance rates, the realities of health and economic disparities (many of these correlated with race) and escalating healthcare/insurance costs pose a major threat to the U.S. economy and to the health of millions of un-/underinsured Americans [55,56,59].

Insurers use ICD-9 codes [60] for clinical diagnoses of health conditions and diseases to assess coverage for hospital and clinic patients, pre-existing conditions, etc. Patient waiver of their HIPAA rights during a clinic visit for release of their health information to other health care providers and to insurance companies are routine and allow the insurance company to decide on the level of coverage for each diagnosis and treatment. Major insurers are members of the national E-Health Initiative . Furthermore, several insurers are discussing the linkage of records across systems [61]. On the surface, this step would be a positive move to contribute towards NHIN. However, there are serious civil liberties concerns with respect to an individual's health information being tracked by insurers throughout their life, particularly with loss of benefits for pre-existing conditions if a person shifts from one insurance provider to another. Furthermore, the rise of genomics and the ability to test for an ever-expanding array of genetic conditions (see above) raises concerns over loss of insurance for pre-existing genetic conditions and newly discovered conditions [62-68]. The sharing of health records of patients between insurance underwriters tends to go against the philosophy of patient/parent control over records in the All Kids Count children's EHR initiatives [27]. In 2007, the U.S. Congress attempted to pass legislation prohibiting genetic discrimination by employers and insurers, although political resistance slowed progression of the legislation, which included a controversial exemption for U.S. military personnel [69,70].

Nevertheless, insurers are playing strongly positive and major roles in EHR development. For example, one insurance company is partnering with a major medical school to evaluate the effectiveness of EHR in reducing pharmacy errors, one of the leading causes of medical errors, thereby improving patient safety [71]. Besides insurers, providers of healthcare services and products have become involved with EHR as well to promote this patient safety focus [72].

### Consortium Activities

Other groups have focused upon the technical aspects of linking, matching and de-duplicating case records in EHR while at the same time generating business case models for specific applications in given medical/health records scenarios. Among the major organizations pursuing these activities include the Public Health Data Standards Consortium (PHDSC; ), which interfaces with the other projects described above to facilitate models for effective EHR implementation.

#### Public Health Data Standards Consortium

PHDSC [73] identified key public health domains, stakeholders in these domains, core public health functions and services, and central data providers and mapped these domains to each other. The primary goal of this effort was to develop a functional model for the EHR that utilizes HL7 messaging standards and that can be demonstrated to link public health agencies with hospitals and other sites of clinical care [73]. Key barriers to EHR adoption are cited, including widespread resistance to computerization of clinics and health care agencies, data silo issues, public perceptions of electronic records and privacy issues (as outlined above with genetic testing), legal and regulatory issues, and most importantly, technical issues in linking and managing large-scale health data systems that use different software, hardware, and coding systems and that are continuously evolving [73]. They provide many case examples in both storybook and extensive formats, mapping the central domains and functions of public health and clinical services/data collection to scenarios ranging from hazardous materials exposure to chronic hypertension, in the process demonstrating the importance of common messaging standards and data sharing agreements to enhance the mission of public health [73].

Similarly, PHDSC [73,74] also addressed applicability of EHR to NHIN, specifically providing an HL7-based model [74] for interoperability between birth hospitals and state health departments, a process that is already well developed in some states (e.g., Rhode Island, as described above). With the various barriers to EHR that have been so far discussed across the various projects described above, PHDSC [73] illustrates that the development of a National Health Information Network (NHIN) is in its infancy, with current development occurring very slowly in the lag phase of an early Verhulst-Pearl logistic growth model [74-76].

#### Public Health Informatics Institute – All Kids Count and Connections

To complement the work of PHDSC and its affiliate organizations (e.g., American Health Information Management Association, National Association of Health Data Organizations), the RWJ-funded All Kids Count program [9,10,25-27] described above has evolved into a new role: supporting and providing models of EHR excellence in data linking and production of functioning systems [77]. This new program, entitled the Connections Community of Practice, continues to be based at the Public Health Informatics Institute (Decatur, GA), but it has carried forward its All Kids Count state model projects into a forum that also includes Regenstrief, Volunteer e-Health, insurance companies, and PHDSC. The goal is for each of the players in the development of EHR to learn freely from each other to expand electronic health records in conjunction with NHII/NHIN. Case examples of state projects to link together different health records, merging data, and dealing with technical problems such as common identifiers for linkage and probabilistic matching of records with poorly matching identifiers are described in this comprehensive report [77].

One of the major achievements of PHDSC and PHII is the establishment of networking among the many diverse groups conducting EHR projects. These organizations have brought projects and institutions out of their "data silos" (i.e., refusal to share data) and fear of HIPAA laws/regulations (see below) to discuss successes and problems in generating linked electronic health records. They have produced fruitful interactions between the state CHP projects, RHIOs, and private EHR projects. One strong resource from these collaborations has been a PHII guidebook [77] for data linkage and records de-duplication, two of the most pressing logistical problems facing all of the EHR projects. Nevertheless, the various EHR projects and consortia must deal with continuing data silo issues and the need to more strongly involve patient advocacy groups in planning and implementation.

### Contextual Environment of EHR – Patient Rights/Advocacy and Legal Issues

In the United States, the legal aspects of sharing data center about two American laws: (a) the Family Educational Rights and Privacy Act (FERPA; Buckley Amendment Title IX of the Higher Education Amendments of 1972; 20 U.S.C. §1232g, 34 CFR 99), and (b) the Health Insurance Portability and Accountability Act (HIPAA; 1996 Amendment Part 7 to Title I of the Employee Retirement Income Security Act of 1974; 42 U.S.C. 1320d-1320d-8, Public Law 104–191, sections 262 and 264).

#### HIPAA

Most public and professional attention has been placed upon the HIPAA, which prohibits health care entities and individuals working for those entities from disclosing any health-related information about a patient without authorization from the patient or patient's legal representative. HIPAA further prohibits discrimination against patients by insurers for pre-existing conditions if the patient has been insured for at least 18 months. Consequently, patient's are given a HIPAA explanatory form and a waiver form for their signature prior to receipt of clinical services, thus allowing the clinical provider to bill the patient's insurance company and, in the process, transmit the patient's health information for the insurer to decide upon the claim and insurance benefit coverage for the patient. HIPAA applies to any type of individual protected health information (PHI), written, verbal, or electronic, and it stipulates stiff legal and financial penalties for violators.

#### FERPA

Similarly, FERPA was established to protect the confidentiality of children's educational records, wherein a child's parent or legal guardian must authorize any release of the child's school records if any agency or individual requests those records; at the age of 18 and beyond, the individual must provide authorization. Since there is overlap between health and education records in many public health databases (e.g., cognitive development, school immunization records), there has been much discussion on the subject of which law takes priority. The general consensus indicates that FERPA overrules HIPAA, in that individuals or their guardians/legal representatives must consent to the sharing of PHI, including health and educational records when both types of information are present. Such consent directly impacts large-scale electronic health records networks at the state level (e.g., Rhode Island) and with the massive RHIOs (e.g., Regenstrief, Volunteer e-Health). Many of these programs are using legally-binding inter-institutional data sharing agreements to allow authorized providers at participating institutions to access data. Furthermore, data security systems include audit trails to monitor any excessive or unauthorized accessions to individual patient records.

#### Rights and Legal Issues

The legal environment of electronic health information has complicated the development of EHR. Rosenbaum et al. [78] describes major health legal issues, specifically addressing the ownership of health information, disclosure of PHI, extent and power of involvement of health insurers, private civil litigation and access to PHI, access to PHI by the government and law enforcement agencies, and basic research access to patient records. Their study compiled interpretations of a broad-based government, academic, health, business, and other private sector experts on EHR and the law. Overall, they agreed that HIPAA generally is designed to prevent abuses of patient confidentiality and discrimination, although certain areas of disagreement exist with respect to HIPAA overruling less stringent state standards for protecting PHI. Additionally, the ownership of EHR (e.g., patient, hospital, insurance companies) remains a major unresolved issue [78]. Rosenbaum et al. [78] provided various models of centralized and decentralized data sharing agreement models, including Regenstrief as a decentralized model. The Robert Wood Johnson Foundation [[58], pp. 98–100] provides a summary of the confusion surrounding HIPAA and states' use of EHR, misunderstandings that have needlessly limited states' implementation of EHR.

## Conclusion

Electronic health records promise to improve health care delivery to every American citizen, reduce medical errors, and make health services more cost effective, while allowing tracking of individuals with serious health conditions by providers. Nevertheless, the development and adoption of this technology has taken on multiple forms and various levels, with simultaneous wide variation in the degree of adoption/use of the technology. Many hospitals, local health departments, and large private clinics are beginning to adopt and use EHR, and the U.S. Health Resources Services Administration (HRSA) has started disseminating funds to smaller health venues, with $31.4 million awarded to 46 regional health center projects in 2007 [79]. Still, the process is gaining momentum but lags without consistent models and a true national effort as compared to well developed national EHR systems in other nations [58,80,81].

The Robert Wood Johnson Foundation [58] cites World Health Organization data indicating that Denmark, England, Finland, the Netherlands, Norway, Sweden, Australia, and New Zealand each have greater than 90% EHR use by ambulatory care providers. These successful national programs have utilized government funding, incentives for physicians to purchase computers and/or secure internet communications, health insurance cards and records having unique identifiers, and successful programs promoting e-referrals and e-prescriptions to pharmacies. In several instances, such programs have been successful with only a small percentage of the government health budget.

In contrast, the United States has broadly funded an array of competing EHR projects [9,10,20-22,45-47] that followed similar pathways but only started serious collaborations on common project issues in 2005 with consortia activities such as the PHII Connections Community of Practice. The United States remains committed to EHR with renewed funding during 2009–2011 as part of its economic stimulus efforts [8]. It remains to be seen if this renewed EHR effort will address the disparate regional, state, and local projects that, ironically, need to be inter-communicating health data. In many ways, the many EHR projects have replicated data silos at a different information level (inter-systems) while attempting to solve data linkage (intra-systems). There have been situations where multiple but complementary EHR projects have been conducted in single states without project leaders being aware of each other. Instead of a national focus, the current U.S. EHR environment represents a selectionist economic/evolutionary model, where many projects are succeeding at some level, but only a few projects will demonstrate the flexibility and scope to promote patient safety and the healthcare needs of both patients and providers.

The Volunteer e-Health RHIO is in the process of attempting to address this inter-systems communication problem. If the Volunteer e-Health RHIO model succeeds, as the Regenstrief RHIO and Rhode Island Kidsnet CHP appear to be doing, then the United States will have three roughly parallel models to follow. The new EHR funding included in the American Recovery and Reinvestment Act [8] probably can be most efficiently used to promote a genuine National Health Information Network (NHIN) [6]. Funding should address inter-systems communication and promotion of health provider use, per the successful international programs in Europe and Australia/New Zealand. There will be no need to fund more experimental programs, only funding to implement RHIOs on a national scale and to research patient safety and system efficiencies for these programs.

To summarize, the complexities of the U.S. health care system and the lack of socialized medicine have hindered the EHR developmental process compared to more streamlined approaches in other nations. Major problems for the development of NHIN include the following issues:

1. Will the EHR remain consumer controlled, or will the system become dominated by commercial interests (e.g., for-profit hospitals, insurance companies) to deny services or charge exorbitant fees to at-risk individuals and persons with disabilities?

2. Similarly, will the primary purpose of EHR be better health/patient safety or better billing for services?

3. Many health care entities, even within systems, continue to operate with data silos and refuse to link patient data.

4. There exists disconnectedness of repetitive systems and systems unawareness of each other, even within the same state (e.g., five projects in Tennessee that have sketchy collaborations, although it seems certain that Volunteer e-Health will emerge as a national model).

5. With limited national and state budgets, many healthcare systems operate with outdated modalities (e.g., DOS systems) and refuse to network even within certain public health departments.

6. Many providers are reluctant to use EHR, instead relying on facsimile and telephone communications of patient conditions, because it has "always" worked fine, despite Hinman's [9] estimates to the contrary.

7. Many hospitals and other providers utilize private vendors for data sourcing, thereby creating potential ethical problems with respect to access to patient data by these private systems.

8. The legality of data sharing and patient consent via FERPA and HIPAA should not be a problem, although non-cooperative providers continue to misconstrue these laws as a smokescreen for data silo issues.

9. Technical issues include linking databases without unique identifiers and the need for extensive, laborious manual verification of non-matching records even with sophisticated deterministic and probabilistic data matching schemes.

10. There is a need to address patient trust and consent given fears of inappropriate data use for research, disclosure to unauthorized individuals, access by insurance companies for denial of coverage for conditions and future coverage, and identity theft.

11. Several confidentiality issues exist, such as a scenario where one database has natural parent information and the linked data has adopted parent information, thereby potentially breaching confidentiality of natural parent information.

12. The incorporation of genomic data on an individual level in these databases, as the Volunteer e-Health Initiative and Regenstrief projects envision, will further affect the size and security of EHR databases [65,82,83].

These are but a few of the complicated issues that have emerged with the convergence of medical technology, information technology, and the psychosocial/political aspects of human societies, in this case, a major Western nation with nearly 300 million people.

Whereas there will be some dissension on EHR efficacy, the overwhelming majority of citizens and healthcare providers will accept and use EHR once appropriate safeguards (e.g., improved data encryption and transmission) and convincing demonstrations are provided to the public. For logistical issues in linking electronic records, the various projects have overcome most of these obstacles (mainly remnants of pre-electronic record-keeping) and can move proactively towards universal user systems.

For the legal environment, the U.S. government needs to clarify the patient privacy rule in HIPAA that allows authorized health provider communications on patient data, de-identification of patient records for research purposes, and reinforcement of patient confidentiality throughout the process. Finally, the CHP and All Kids Count programs have stressed patient control over records, a model that hopefully will be broadly adopted to deter employer, insurance, provider, and other forms of discrimination.

The projects described here include many optimists whose goal is to improve public health. Furthermore, the national consortia of many different interest groups are converging on common themes and technological standards that should produce an eventual working NHIN, most likely not within ten years, although the major players and technical pieces are gradually coming together.

## Abbreviations

CHP: Child Health Profile; CHI: Consolidated Health Informatics; CPT-4: Current Procedural Terminology Version 4; CSHCN: Children with Special Health Care Needs; DOS: Disk Operating System; DPT: Diphtheria Pertussis Tetanus immunization; EHR: Electronic Health Record; FERPA: Family Educational Rights and Privacy Act; HIPAA: Health Insurance Portability and Accountability Act; HIT: Health Information Technology; HL7: Health Level 7; HRSA: Health Resources Services Administration; ICD-9: International Classification of Diseases Version 9; IOM: Institute of Medicine; LOINC: Logical Observation Identifiers Names and Codes; NBS: Newborn Screening (Genetic and Metabolic Conditions); NCPDP SCRIPT: National Council for Prescription Drug Programs preSCRIPTion coding standards; NHII: National Health Information Infrastructure; NHIN: National Health Information Network; PHII: Public Health Informatics Institute; PHDSC: Public Health Data Standards Consortium; RHIO: Regional Health Information Organization; RWJ: Robert Wood Johnson Foundation; TMS: Tandem Mass Spectrometry; WIC: Women Infants Children program.

## Competing interests

The author received partial funding from GlaxoSmithKline for an undergraduate medical education teamwork training project that was unrelated to this study.

## Authors' contributions

The author completed all aspects of the article as original works.

## References

[B1] Murray CJL, Kulkarni SC, Michaud C, Tomijima N, Bulzacchelli MT, Iandiorio TJ, Ezzati M (2006). Eight Americas: Investigating mortality disparities across races, counties, and race-counties in the United States. PLoS Med.

[B2] Kohn LT, Corrigan JM, Donaldson MS, Eds (2000). To err is human: building a safer health system.

[B3] Smedley BD, Stith AY, Nelson AR, Eds (2003). Unequal treatment: Confronting racial and ethnic disparities in health care.

[B4] IOM Committee on Quality of Health Care in America (2001). Crossing the quality chasm: A new health system for the 21^st ^century.

[B5] Davis K, Schoen C, Schoenbaum SC, Doty MM, Holmgren AL, Kriss JL, Shea KK (2007). Mirror, mirror on the wall: an international update on the comparative performance of American health care.

[B6] Yasnoff WA, Humphreys BL, Overhage JM, Detmer DE, Brennan PF, Morris RW, Middleton B, Bates DW, Fanning JP (2004). A consensus action agenda for achieving the National Health Information Infrastructure. J Am Med Inform Assoc.

[B7] Ford EW, Menachemi N, Phillips MT (2004). Predicting the adoption of electronic health records by physicians: when will health care be paperless?. J Am Med Inform Assoc.

[B8] (2009). American Recovery and Reinvestment Plan of 2009. Congressional Record – US House of Representatives, H1307–H1516.

[B9] Hinman AR, Eichwald J, Linzer D, Saarlas KN (2005). Integrating child health information systems. Am J Public Health.

[B10] Linzer DS, Lloyd-Puryear MA, Mann M, Kogan MD (2004). Evolution of a child health profile initiative. J Public Health Manag Practice.

[B11] McPherson M, Arango P, Fox H, Lauver C, McManus M, Newacheck PW, Perrin JM, Shonkoff JP, Strickland B (1998). A new definition of children with special health care needs. Pediatrics.

[B12] van Dyck PC, Kogan MD, McPherson MG, Weissman GR, Newacheck PW (2004). Prevalence and characteristics of children with special health care needs. Arch Pediatr Adolesc Med.

[B13] Huang ZJ, Kogan MD, Yu SM, Strickland B (2005). Delayed or forgone care among children with special health care needs: an analysis of the 2001 National Survey of Children with Special Health Care Needs. Ambul Pediatr.

[B14] U.S. Department of Health and Human Services (2000). Healthy People 2010 Washington, DC.

[B15] Arias E (2002). United States life tables, 2000. National Vital Statistics Reports.

[B16] Sutton PD, Matthews TJ (2004). Trends in characteristics of births by state: United States, 1990, 1995, and 2000–2002. National Vital Statistics Reports.

[B17] Waisbren SE, Albers S, Amato S, Ampola M, Brewster TG, Demmer L, Eaton RB, Greenstein R, Korson M, Larson C, Marsden D, Msall M, Naylor EW, Pueschel S, Seashore M, Shih VE, Levy HL (2003). Effect of expanded newborn screening for biochemical genetic disorders on child outcomes and parental stress. JAMA.

[B18] Desposito F, Lloyd-Puryear M, Tonniges TF, Rhein F, Mann M (2001). Survey of pediatrician practices in retrieving statewide authorized newborn screening results. Pediatrics.

[B19] American Academy of Pediatrics Newborn Screening Task Force (2000). Serving the family from birth to the medical home – Newborn Screening: A blueprint for the future, a call for a national agenda on state newborn screening programs. Pediatrics.

[B20] Hollar D, Copeland MA, Lozzio C, Lainhart R, Wilson B, Blake T, Fleshood L Tennessee Child Health Profile (TN-CHP): An integrated data warehouse serving children with special health care needs. Proceedings of the 132nd Annual Meeting of the American Public Health Association: 6–10 November 2004; Washington, DC.

[B21] March of Dimes (2003). Maternal, infant, and child health in the United States, 2003. Washington, DC.

[B22] Swanson R Protecting Michigan's children using the Michigan Childhood Immunization Registry (MCIR). Proceedings of All Kids Count: Developing Child Health Information Systems to meet Medical Care and Public Health Needs: 3–4 December 2003.

[B23] Schulze A, Lindner M, Kohlmuller D, Olgemoller K, Mayatepek E, Hoffmann GF (2003). Expanded newborn screening for inborn errors of metabolism by electrospray ionization-Tandem Mass Spectrometry: results, outcome, and implications. Pediatrics.

[B24] Green NS, Dolan SM, Murray TH (2006). Newborn screening: Complexities in universal genetic testing. Am J Public Health.

[B25] Public Health Informatics Institute (2004). Rhode Island: Confidentiality, the challenge of time, growth and change – Factors triggering reassessment for integrated information systems. Connections: Creating a road map – Sharing knowledge about integrating child health information systems Decatur, GA.

[B26] Fehrenbach SN, Kelly JCR, Vu C (2004). Integration of child health information systems: Current state and local health department efforts. J Public Health Manag Pract.

[B27] Hinman AR, Atkinson D, Diehn TN, Eichwald J, Heberer J, Hoyle T, King P, Kossack RE, Williams DC, Zimmerman A (2004). Principles and core functions of integrated child health information systems. J Public Health Manag Practice.

[B28] Levy HL, Sepe SJ, Shih VE, Vawter GF, Klein JO (1977). Sepsis due to *Escherichia coli *in neonates with galactosemia. N Eng J Med.

[B29] Greenberg CR, Dilling LA, Thompson R, Ford JD, Seargeant LE, Haworth JC (1989). Newborn screening for galactosemia: a new method used in Manitoba. Pediatrics.

[B30] Burton BK (1998). Inborn errors of metabolism in infancy: a guide to diagnosis. Pediatrics.

[B31] D'Alessandro DM, Dosa NP (2001). Empowering children and families with information technology. Arch Pediatr Adolesc Med.

[B32] Sia C, Tonniges TF, Osterhus E, Taba S (2004). History of the medical home concept. Pediatrics.

[B33] Strickland B, McPherson M, Weissman G, van Dyck P, Huang ZJ, Newacheck P (2004). Access to the medical home: results of the National Survey of Children with Special Health Care Needs. Pediatrics.

[B34] Cooley WC, McAllister JW (2004). Building medical homes: Improvement strategies in primary care for children with special health care needs. Pediatrics.

[B35] Hastings TM (2004). Family perspectives on integrated child health information systems. J Public Health Manag Practice.

[B36] Gurian EA, Kinnamon DD, Henry JJ, Waisbren SE (2006). Expanded newborn screening for biochemical disorders: the effect of a false-positive result. Pediatrics.

[B37] Tarini BA, Christakis DA, Welch HG (2006). State newborn screening in the tandem mass spectrometry era: More tests, more false-positive results. Pediatrics.

[B38] Hollar D, Lozzio C, Lemak R, Eubanks RL, Evans M Key elements in partnership and infrastructure development for electronic child health profiles: a description of two parallel, complementary projects. Proceedings of the 133rd Annual Meeting of the American Public Health Association, 9–14 December 2005; Philadelphia.

[B39] Wild EL, Fehrenbach SN (2004). Assessing organizational readiness and capacity for developing an integrated child health information system. J Public Health Manag Pract.

[B40] Wild EL, Hastings TM, Gubernick R, Ross DA, Fehrenbach SN (2004). Key elements for successful integrated health information systems: Lessons from the states. J Public Health Manag Practice.

[B41] Slagle TA (1999). Perinatal information systems for quality improvement: Visions for today. Pediatrics.

[B42] Handler A, Grason H, Ruderman M, Issel M, Turnock B (2002). Assessing capacity and measuring performance in maternal and child health. Mat Child Health J.

[B43] Beal AC, Co JPT, Dougherty D, Jorsling T, Kam J, Perrin J, Palmer RH (2004). Quality measures for children's health care. Pediatrics.

[B44] Johnson KB (2001). Barriers that impede the adoption of pediatric information technology. Arch Pediatr Adolesc Med.

[B45] Biondich PG, Grannis SJ (2004). The Indiana Network for Patient Care: an integrated clinical information system informed by over thirty years of experience. J Public Health Manag Pract.

[B46] McDonald  CJ, Overhage  JM, Barnes M, Schadow  G, Blevins L, Dexter PR, Mamlin B (2005). The Indiana Network for Patient Care: a working local health information infrastructure. Health Affairs.

[B47] Goetz MD, Frisse ME (2004). Technical discussion: state and regional demonstrations in health information technology. Nashville, TN, Vanderbilt Center for Better Health.

[B48] Annie E, Casey Foundation (2004). Kids Count Data Book Baltimore, MD.

[B49] Sankaran V The Consolidated Health Informatics Initiative and the Functioning and Disability Domains. Proceedings of the Interagency Committee on Disability Research State of the Art Conference: New Federal Applications of the ICF: 10–11 July 2007; Washington, DC.

[B50] Consolidated Health Informatics. http://www.hhs.gov/healthit/chi.html.

[B51] (2005). Giving more than a nod to the wave of the future. Manag Care.

[B52] Leatherman S, Berwick D, Iles D, Lewin LS, Davidoff F, Nolan T, Bisognano M (2003). The business case for quality: Case studies and an analysis. Health Affairs.

[B53] Well A (2007). Next steps for Tennessee: A conversation with Gov. Phil Bredesen. Health Affairs.

[B54] (2007). Missouri, Tennessee Medicaid changes endangering health. The Nation's Health.

[B55] Robert Wood Johnson Foundation (2007). Comparing federal government surveys that count uninsured people in America. Princeton, NJ.

[B56] Congressional Budget Office (2003). How many people lack health insurance and for how long? Economic and budget issue brief. Washington, DC.

[B57] Governor's Communications Office (2006). Bredesen appoints members to ehealth advisory council. Nashville, TN.

[B58] Robert Wood Johnson Foundation (2008). Health information technology in the United States: Where we stand, 2008.

[B59] Krieger N (2005). Stormy weather: Race, gene expression, and the science of health disparities. Am J Public Health.

[B60] World Health Organization (2000). The International Classification of Diseases, 9^th ^edition (ICD-9). Geneva.

[B61] Pack T (2006). Insurers set to put patient files on the web: records to follow patients if job, insurer changed.

[B62] Apse KA, Biesecker BB, Giardiello FM, Fuller BP, Bernhardt BA (2004). Perceptions of genetic discrimination among at-risk relatives of colorectal cancer patients. Genetics in Medicine.

[B63] Burke W, Zimmern RL (2004). Ensuring the appropriate use of genetic tests. Nature Reviews Genetics.

[B64] Genetics and Public Policy Center (2004). Prenatal genetic testing technology: Science, policy, and ethics – Testimony of Kathy Hudson, PhD, Director, Genetics and Public Policy Center before the United States Senate Committee on Commerce, Science, and Transportation, Subcommittee on Science, Technology, and Space, 17 November 2004.

[B65] Genetics and Public Policy Center (2007). U.S. public opinion on uses of genetic information and genetic discrimination.

[B66] Lin Z, Owen AB, Altman RB (2004). Genomic research and human subject privacy. Science.

[B67] Low L, King S, Wilkie T (1998). Genetic discrimination in life insurance: Empirical evidence from a cross sectional survey of genetic support groups in the United Kingdom. BMJ.

[B68] Rothstein MA (2004). Genetics and life insurance: Medical underwriting and social policy.

[B69] Wadman M (2007). US genetics bill blocked again. Nature.

[B70] (2007). Pulling rank: why should US military personnel be singled out for genetic discrimination?. Nature.

[B71] Robert Wood Johnson Foundation (2007). University of Miami, Humana partner for medication safety research. Princeton, NJ.

[B72] Robert Wood Johnson Foundation (2007). Ohio health care company to award $1 million for care quality, patient safety improvement initiatives. Princeton, NJ.

[B73] Public Health Data Standards Consortium Ad Hoc Task Force on Electronic Health Record – Public Health (2004). White paper – Electronic health records: Public health perspectives. Baltimore, MD.

[B74] Public Health Data Standards Consortium (2005). Response to the request for information on the development and adoption of a National Health Information Network from the Office of the National Coordinator for Health Information Technology, Department of Health and Human Services. Baltimore, MD.

[B75] Verhulst PF (1838). Notice sur la loi que la population pursuit dans son accroissement. Correspondance mathématique et physique.

[B76] Pearl R, Reed LJ (1920). On the Rate of Growth of the Population of the United States Since 1790 and its Mathematical Representation. Proc Natl Acad Sci USA.

[B77] Public Health Informatics Institute (2006). Connections – The unique records portfolio: A guide to resolving duplicate records in health information systems. Decatur, GA.

[B78] Rosenbaum S, Borzi PC, Repasch L, Burke T, Benevelli JF (2005). Charting the legal environment of health information. Proceedings of Legal Environment for Emerging Information Systems and Implications for Access to Quality and Disparities Data: 26 October 2004.

[B79] Health Resources Services Administration (2007). HRSA awards $31.4 million to expand use of health information technology at health centers. Washington, DC.

[B80] Lium J-T, Lærum H, Schulz T, Faxvaag A (2006). From the front line, report from a near paperless hospital: Mixed reception among health care professionals. J Am Med Inform Assoc.

[B81] Schade CP, Sullivan FM, de Lusignan S, Madeley J (2006). e-Prescribing, efficiency, quality: Lessons from the computerization of UK family practice. J Am Med Inform Assoc.

[B82] Levy S, Sutton G, Ng PC, Feuk L, Halpern AL, Walenz BP, Axelrod N, Huang J, Kirkness EF, Denisov G, Lin Y, MacDonald JR, Pang AWC, Shago M, Stockwell TB, Tsiamouri A, Bafna V, Bansal V, Kravitz SA, Busam DA, Beeson KY, McIntosh TC, Remington KA, Abril JF, Gill J, Borman J, Rogers Y-H, Frazier ME, Scherer SW, Strausberg RL, Venter JC (2007). The diploid sequence of an individual human. PLoS Biol.

[B83] Safrin S (2004). Hyperownership in a time of biotechnological promise: the international conflict to control the building blocks of life. Am J Int Law.

